# A Systematic Review to Investigate Whether Angiotensin-(1-7) Is a Promising Therapeutic Target in Human Heart Failure

**DOI:** 10.1155/2013/260346

**Published:** 2013-12-12

**Authors:** Vincent C. H. Lee, Elizabeth N. Lloyd, Helena C. Dearden, Kenneth Wong

**Affiliations:** ^1^Hull York Medical School, Castle Hill Hospital, Castle Road, Kingston upon Hull HU16 5JQ, UK; ^2^Department of Cardiovascular and Respiratory Studies, Hull and East Yorkshire Medical Research and Teaching Centre, Daisy Building, Castle Hill Hospital, Castle Road, Kingston upon Hull HU16 5JQ, UK

## Abstract

*Context*. Heart failure (HF) is a common condition causing much morbidity and mortality despite major advances in pharmacological and device therapies. Preclinical data suggest a cardioprotective role of Angiotensin-(1-7) in animal models of HF. *Objective*. Perform a systematic review on the effects of Angiotensin-(1-7) on humans, focusing on HF. Results. 39 studies were included in the review (4 in human HF and (35) in non-HF patients). There is only one intervention study on 8 patients with human HF, using Angiotensin-(1-7), with forearm blood flow (FBF) as the endpoint. Angiotensin-(1-7) caused no significant effect on FBF in this HF study but caused vasodilation in 3 out of 4 non-HF studies. In one other non-HF study, Angiotensin-(1-7) infusion led to a significant increase in blood pressure in normal men; however, effects were <0.03% that of angiotensin II. Cardioprotective effects seen in non-HF studies include for instance beneficial actions against atherosclerosis and myocardial fibrosis. *Conclusions*. The main finding of our systematic review is that Angiotensin-(1-7) plays an important cardioprotective role in HF in animals and in patients without heart failure. More research is required to test the hypothesis that Angiotensin-(1-7) benefits patients with heart failure.

## 1. Introduction

Heart failure (HF) is a major health problem for many developed world populations and has a relatively poor prognosis. Within the US population, the incidence approaches 1%, with a lifetime risk of 1 in 5 for both men and women at the age of 40 years [1]. In 2005, there was an estimated near 1.1 million admissions to American hospitals associated with HF, which was up from approximately 400,000 in 1979 [2]. This substantial increase in prevalence and hospitalisation meant an epidemic was declared [3, 4]. Over the coming years, the prevalence is likely to increase due to changing lifestyles and diets of the developing world, advances in heart failure therapeutics, an increase in prevalence of conditions which have cardiovascular consequences such as obesity and diabetes, and better survival from other heart conditions where HF is the end stage especially with the increased use of primary angioplasty for myocardial infarction. Figures from the United States show an estimated bill totalling $39.2 billion in 2010, to cover the direct and indirect cost of HF [5]. One in 8 death certificates in America mentioned HF, and in 20% of cases, it was the primary cause. In 2006, the number of any-mention deaths from HF was 283,000 [1]. Five-year mortality is 45–60% [6], and hospitalisations increased in the months prior to their death [7]; moreover, after the first hospitalisation, the 5-year mortality was greater than 75% [8]. This deadly syndrome, where the renin-angiotensin-aldosterone system (RAAS) is of great importance, is characterised by dyspnoea, venous congestion, and oedema, the consequences of anatomical and functional defects within the heart.

### 1.1. Angiotensin-(1-7): The Peptide

This bioactive endogenous heptapeptide is taking increasing amounts of interest from investigators because of its potential as a therapeutic agent. Angiotensin-(1-7) (Ang-(1-7)) is becoming recognised as an increasingly important player of the RAAS where accumulating evidence suggests that it may have a key role in the regulation and homeostasis of the RAAS, exerting cardiovascular protection. Ang-(1-7) is different from angiotensin II due to the absence of an amino acid at position 8, described as the most pleiotropic metabolite of angiotensin I (Ang I), and is known to have actions which on most occasions are opposite (but may be identical) of those known for angiotensin II (Ang II) [9, 10]. Many effects of Ang-(1-7) are mediated via the high-affinity G protein-coupled receptor Mas, where Ang-(1-7) is an endogenous ligand [11]. Endopeptidases (such as neutral endopeptidase (NEP)) and angiotensin-converting enzyme-2 (ACE2) contribute to the alternative pathways of Ang-(1-7) generation, using the substrates angiotensin I (Ang I) and angiotensin II (Ang II), respectively [12–14], whereas it has been shown that Ang-(1-7) is degraded to angiotensin- 1-5 (Ang-(1-5)) by angiotensin-converting enzyme (ACE) [15]. In contrast, ACE has also been shown to generate Ang-(1-7) from angiotensin-(1-9) [16]. Ang-(1-7) studies have shown that it is a potential endogenous counterregulator of the RAAS cascade [15, 17]. Physiological concentration of Ang-(1-7) in man is thought to be 10 pmol/L [18, 19]. In 1988, Schiavone et al. suggested that Ang-(1-7) peptide was indeed biologically active [20], raising the possibility of potential benefits of Ang-(1-7).

### 1.2. Preclinical Studies

There has been an abundance of animal studies investigating the effects of Ang-(1-7) in a variety of environments. Increased activation of the RAAS in heart failure is a compensatory mechanism. However, this increased afterload can have adverse effects on cardiac function. Angiotensin converting enzyme inhibitors are commonly seen in the management plan of HF; one reason is to reduce arterial pressure and to reduce the levels of Ang II. Vasodilatation is a property of Ang-(1-7) [21]. It does so by various postulated mechanisms such as releasing nitric oxide (NO) and prostaglandins [22–25]. Both Brosnihan et al. [26] and Porsti et al. [27], by administering NO synthase inhibitors, suggested that the vasodilatory effect of Ang-(1-7) may be at least partly dependent on the release of NO. Furthermore, Benter et al.'s work would suggest that Ang-(1-7) might oppose the haemodynamic actions of Ang II. Another peptide contributing to the vasodilatory effects of Ang-(1-7) is bradykinin (BK) [29], a molecule Ang-(1-7) is shown to interact with. Paula and colleagues [30] suggested that Ang-(1-7) potentiates the hypotensive effect of bradykinin *in vivo* and prostaglandins may participate in the mechanism of potentiation by Ang-(1-7).

There are other potentially cardioprotective effects of Ang-(1-7) shown in animal studies.

### 1.3. Aims and Objectives

The objective of this systematic review will be to discuss human studies on the beneficial effects of angiotensin-(1-7) focusing on patients with heart failure.

## 2. Methods

### 2.1. Search Strategy of the Systematic Review

Highly sensitive search strategies were developed using appropriate subject headings and text word terms. The following electronic databases were searched: Pubmed, Embase, and Cochrane. In addition, conference proceedings and reference lists of all included studies were scanned to identify additionally potentially relevant studies. There were no start year restrictions, but the studies examined were restricted to English language reports.

### 2.2. Data Extraction

Two reviewers screened the titles (and abstracts if available) of all reports identified by the search strategy. Full copies of potentially relevant reports were obtained, studied, and assessed for inclusion. Data was discussed with the senior author, and disagreements were resolved by consensus.

## 3. Results

**Figure 1:**
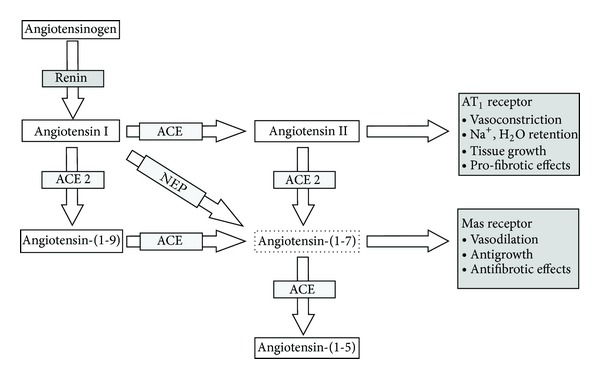
Demonstrating the relationship between Angiotensin I, Angiotensin II, Ang-(1-7), and the converting enzymes (ACE and ACE2).

**Figure 2:**
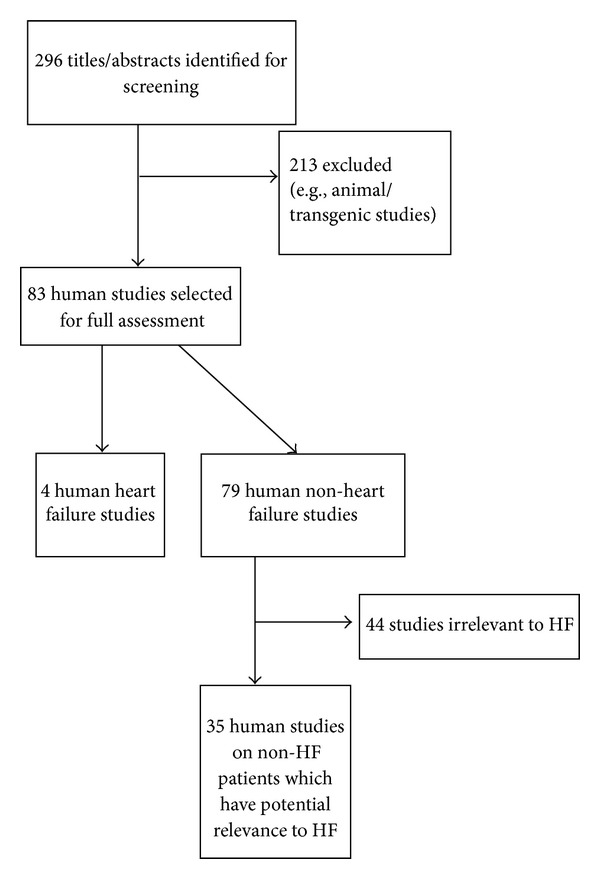
Flow of studies through the review.

Figure 1 helps the reader appreciate the close relationship between Angiotensin-(1-7) and Angiotensin II.  Figure 2 summarises the types of studies considered in the review.

### 3.1. Studies on Human Heart Failure Patients

See Table 1.

### 3.2. Studies on Non-Heart-Failure Patients

See Table 2.

## 4. Discussion

The main finding of this systematic review is that Ang-(1-7) plays an important cardioprotective role in heart failure in animals and in patients without heart failure. However, to date, the evidence for the role of Ang-(1-7) in human heart failure is limited (Table 1), whilst there are many more studies in non-heart-failure patients (Table 2).

The only study which evaluated the effects of Ang-(1-7) on patients with heart failure examined only 8 patients. These patients were all treated with an ACE inhibitor. The interaction of Ang-(1-7) with bradykinin was elegantly studied [31]. Ang-(1-7) did not have any significant effect on the pulse rate, blood pressure, or forearm blood flow in the noninfused arm during infusion of the peptide. Slight vasoconstriction was observed during Ang-(1-7) infusion at 500 pmol/min and 5000 pmol/min; however, this was not statistically significant, and the absolute magnitude of the effect was very small. Importantly, no effect was observed with lower or higher doses. When Ang-(1-7) was coinfused with BK, the results were very similar to the initial infusion of BK alone. Interestingly, it showed a slight reduction in response to BK, though this was not statistically significant. Thus, intriguingly, the results from this study [31] are contradictory to the wealth of evidence gained from animal studies. Nevertheless, the findings were similar to another small study conducted on 8 non-heart-failure subjects [19]. Another study also found that if Ang-(1-7) was administered above a certain concentration (dose), its actions were abolished [37].

It is important to bear in mind the species gap between humans and animals and also the differences in methodologies used in the studies (e.g., some animal studies studied the effect of Ang-(1-7) on coronary arteries and others looked at mesenteric arteries). It seems that Ang-(1-7) is biologically inactive, in keeping with a study by Kono et al. [62] who also reported biological inactivity. If anything, its effects appear to have opposite actions (albeit nonsignificantly) to what is generally believed, a vasodilating, antiproliferative, and counterregulatory peptide. This lack of effect of Ang-(1-7) on haemodynamics is intriguing. It should be noted that the study had a relatively small sample size: 8 patients with chronic heart failure secondary to left ventricular dysfunction were recruited. The study could be underpowered statistically. All the patients in the study were already taking ACE inhibitors; therefore, the effect of Ang-(1-7) potentiating BK may have been obscured.

On the other hand, Ang-(1-7) attenuates vasoconstriction and increases blood flow in some studies, involving a larger number of patients, across different vascular beds, including the forearm and internal mammary artery [18, 32, 34, 44]. Thus, there are human studies demonstrating that the vasodilating property Ang-(1-7) is believed to hold. Ueda et al. [18] provided evidence of Ang-(1-7)'s action of attenuating vasoconstriction by Ang II in a dose-dependent manner. Sasaki et al. [63] supported this observation of vasodilation. The findings from Roks et al. [34] and Ueda et al. [44] were also consistent with the detection of a vasodilatory effect of Ang-(1-7). Reference [34] had a study population of 25 patients undergoing CABG (one did not have CABG), and here, Ang-(1-7) was found to behave like an ACE inhibitor. The blockade of vasoconstriction in this study could be due to many mechanisms which include the counterregulation of Ang II signalling [36] and the inhibition of ACE. Reference [44] involved a double-blind crossover design, allowing less room for bias. Coinfusion of Ang-(1-7) at 1000 pmol/min with BK significantly shifted the dose-response curve to the left; this was not the case at 100 and 10 pmol/min (additional analysis was performed to take into account the possible tachyphylaxis of BK in the study, which then found Ang-(1-7) at 100 pmol/min to show effect). Ang-(1-7) seems to operate within a specified range according to a few human studies so far [31, 37, 44, 63]. Wilsdorf et al. [19] result of Ang-(1-7) having no significant effect on BK may have been due to fact that Wilsdorf et al. did not take into account tachyphylaxis in the data; therefore, the effect of Ang-(1-7) may have been masked by this possible phenomenon. References [44, 65] both found tachyphylactic responses to BK. Moreover, Wilsdorf and colleagues [19] did not analyse and measure greater doses of Ang-(1-7), which [44] did and found a significant effect.

Other studies about Ang-(1-7) in human heart failure have provided important insight into its role and suggested mechanisms, whereby it might be further exploited as a peptide with cardioprotective therapeutic benefits. Zisman et al. [12] validated a potential role for Ang-(1-7) in the human failing heart in an elegant study involving explanted hearts. This study involved 22 patients with heart failure due to either end-stage idiopathic cardiomyopathy (IDC) or primary pulmonary hypertension (PPH), compared with 13 patients with normal left ventricles (LV) on echocardiography prior to organ donation, which eventually sadly could not take place due to ABO blood type or donor/recipient size mismatch. In the transplanted hearts, Ang-(1-7) forming activity was significantly increased in the IDC left ventricle; the forming activity was greater for Ang II as a substrate than that for Ang I. Furthermore, the forming activity was greater than 4-fold (Ang II as substrate) compared to the nonfailing left ventricle. Ang II as a substrate for Ang-(1-7) appears to have activity in more areas of the heart than for Ang I, increasing the levels of Ang-(1-7) in both IDC ventricles and the PPH right ventricle. It should be remembered that the PPH left ventricle was not dysfunctional, which may explain the lack of increased Ang-(1-7) forming activity in the left ventricles of patients suffering from primary pulmonary hypertension. ACE inhibitor therapy did not significantly affect angiotensinase activity. Angiotensinase activity was higher in the failing hearts than the non-failing hearts. Zisman et al. [12] indicated a role for neutral endopeptidase and ACE2 in the formation of Ang-(1-7). The Ang-(1-7) forming activity was greater when Ang II was the substrate; moreover, the forming activity was identified in more areas of the heart, suggesting a major role for ACE2 in the failing heart.

Furthermore, few animal studies have shown that AT2 mediates antiproliferative and apoptotic signaling [66]. This has potentially beneficial consequences: it may positively influence the remodelling process known to be detrimental to cardiac function. However, it is unlikely that a direct relation between Ang-(1-7) and the AT2 receptor exists because it is not a natural ligand for it. It may be possible for there to be cross-talk between the Mas receptor and AT2 receptor. There is a strong correlation between Ang-(1-7) and Ang II, being efficiently converted, and the given evidence from previous studies in favour of Ang-(1-7) as a promising peptide to oppose Ang II effects suggests a potential counterregulatory role of Ang-(1-7) in the RAAS, thus being cardioprotective. This study has a few drawbacks; because of the method used to homogenise tissue, influential enzymes may have been lost, therefore unable to exert their effects on either Ang I or Ang II. Not only that, but also, as the authors were focusing on membrane-bound angiotensinases, they may have not identified the potential effects of the soluble ACE2. ACE2 gene expression was shown to be upregulated in the human failing heart in a study by Goulter et al. [67]. Putting the evidence from Zisman et al. [12], Goulter et al. [67], and Pan et al. [35] who declared positive influences by Ang-(1-7) on metalloproteinases (as a measure of myocardial fibrosis in the failing human heart) together, they showed Ang-(1-7) to be a promising physiological peptide in the failing human heart. In the postmyocardial infarction period, an increased matrix metalloproteinase (MMP)/tissue inhibitors of matrix metalloproteinases (TIMPs) ratio contributes to the remodelling stage [68]. Spinale et al. [69] and Schwartzkopff et al. [70] also reported changes in the MMP/TIMP ratio in the human failing heart.

Campbell et al. [13] have importantly provided evidence of Ang-(1-7)'s presence within the coronary sinus and arterial (radial) circulation of patients with heart failure taking ACE inhibitors. Here, an increased level of Ang-(1-7) was shown, by 39- and 22-fold in the coronary sinus and radial arterial blood, respectively, compared to non-heart-failure patients who were also not on ACE inhibitors. Ang-(1-7) appeared to rise in a parallel manner with Ang I levels. These are significant findings as ACE inhibitors now form the cornerstone of the management plan for heart failure. In patients treated with ACE inhibitors, angiotensin I goes up, leading to a rise in Ang-(1-7) via the NEP-like pathway. Thus, the NEP pathway is recognised as an alternate route for the production of Ang-(1-7) [12, 71]. When associated with ACE inhibition, the Ang-(1-7)/Ang II ratio has been found to be increased by 7.5- and 2.25-fold in the coronary sinus and arterial blood respectively. This provides further strong evidence in support of the role of the NEP-like pathway.

As discussed, Ang-(1-7) attenuates vasoconstriction and increases FBF in some studies [18, 34, 44, 63]. Further, Ang-(1-7) has anti-aggregatory effects [37, 43], opposes Ang II signalling in endothelial cells [36], and has beneficial effects against human atherosclerosis [58]. Therefore, finding it within the coronary sinus is highly regarded. However, Davie and McMurray [31] and Wilsdorf et al. [19] did not find any effect on the FBF in a small heart failure cohort and another small study of non-heart-failure patients. Campbell and colleagues [13] have presented data displaying a shift in the balance within the RAAS towards Ang-(1-7), increasing the physiological armoury against the deleterious effects of the ACE-Ang II-angiotensin subtype 1 receptor (AT1R) axis. Lin et al. [41] suggested a positive feedback loop where increased levels of Ang-(1-7) could increase the levels of ACE2 which would further increase Ang-(1-7). This was only shown *in vitro*. The common theme from these two studies along with Zisman et al. [12] is an increased level of Ang-(1-7). The study population of [13] was relatively small, involving 9 patients; however, this study has provided results proposing a beneficial cardioprotective role for Ang-(1-7) in heart failure. The study population was not homogeneous; moreover, there were a number of differences between the two groups, for instance, drug therapy. One possible explanation for the results in the study is that ACE inhibitor therapy can increase Ang-(1-7) levels [46].

Additionally, in 33 patients with end-stage HF undergoing heart transplant, as well as 11 controls, MAS17, MMP3, and collagen I mRNA expression were analysed in myocardial biopsies [32]. The patients were relatively young (mean age 54) but suffered severe left ventricular impairment (mean EF 21%). Their left ventricles were significantly dilated (mean 7.2 cm). Just over half had ischaemic heart failure, and 16 had nonischaemic aetiology. In this study, Perales et al. [32] demonstrated that the level of MMP3 and collagen I expressions was suggestive of the tissue being in the remodelling stage in a proportion of myocardium studied. Importantly, within the same subset of myocardium, there was an increased expression of the Mas receptor. As this receptor has been shown to mediate many actions of Ang II, it suggests a role for Ang-(1-7) in the remodelling process. Interestingly, in the small sample of hearts studied, there was no significant difference between patients with MMP3 expression regarding aetiology, severity of symptoms as measured by NYHA class, medication, or left ventricular dilation, although the abstract (which is as yet unpublished in full) did not specify whether there was any significant effect of ejection fraction on MMP3 expression.

## 5. Clinical Implications and Future Research

Heart failure (HF) is a common condition associated with significant morbidity and mortality despite major advances in medical, revascularization, and modern cardiac resynchronisation therapy (CRT)/implantable defibrillator (ICD) devices [72]. In 2006, more than 400,000 hospitalisations were recognised as due to or complicated by HF, accounting for about 4 million NHS hospital bed-days annually. The UK national HF audit for 2008/2009 showed that 26% of patients aged <75 years and 56% of those aged >75 years would be dead within a year.

Stem cell therapy is the subject of many substantial research programmes. An alternative approach is to stimulate progenitor cells and vascular proliferation. Angiotensin (Ang)-(1-7) is an endogenous ligand for the G protein-coupled receptor Mas [11], which has a pivotal role in preserving normal endothelium-dependent relaxation [73]. Ang-(1-7) infusion stimulated proliferation of endothelial progenitor cells isolated from rodents. The theoretical beneficial effects of Ang-(1-7) in animal research on endothelial progenitor cells need to be further tested in humans. Human studies already suggest that Ang-(1-7) attenuates vasoconstriction, increases FBF in non-heart-failure patients, and opposes Ang II signalling in endothelial cells.

Importantly, Professor Walther's team has also found that infusion of Ang-(1-7) after MI in an animal model increased the number of c-Kit- and vascular endothelial growth factor-positive cells in infarcted hearts, inhibited cardiac hypertrophy, and improved cardiac function [74]. Furthermore, the nonpeptidic Ang-(1-7) receptor agonist AVE0991 also improved cardiac contractility in diabetic rats [75]. In addition, Ang (1-7) stimulates haematopoietic progenitor cells *in vitro* and *in vivo* [76]. Ang-(1-7) has already been studied in phase I/II trials in patients with solid tumours [77, 78]. Daily subcutaneous doses of 2.5–100 micrograms/kg/day were safe and well tolerated. Thus, Ang-(1-7) is a candidate molecule that has already been administered to patients with breast cancer undergoing chemotherapy to correct anaemia (a common comorbidity in HF). In a small study of 8 patients with chronic HF, Ang (1-7) had no significant effect on blood pressure (BP) and caused no adverse effects [31]. Absence of a vasodilator response in patients who are already on ACE inhibitors might be explained at least in part by the fact that the peptide Ang-(1-7) is metabolised by ACE. There is growing evidence to suggest that the beneficial effects of ACE inhibitors and angiotensin receptor blockers (ARBs) are at least in part mediated via Ang-(1-7).

In addition, chronic angiotensin-(1-7) selectively prevents cardiac fibrosis in the DOCA-salt model of hypertension, without any effect on blood pressure or cardiac hypertrophy [79]. Further support of the role of Ang-(1-7) in helping myocardial fibrosis came from observation by Raizada's team that the antifibrotic effect of an ACE2 activator correlated with increased cardiac Ang-(1-7) immunostaining [80]. Metalloproteinases and TIMPs appear to have an important role in myocardial fibrosis and cardiac dysfunction in HF. Pan et al. [35] demonstrated how Ang-(1-7) decreased ratios of MMPs to TIMPs in human cardiac cells. A study performed on patients with heart failure examining the effects of Ang-(1-7) on the expression of MMPs and TIMPs will prove beneficial. The ratios of these enzymes are altered to some degree in HF [69, 70]. This may show direct effects of Ang-(1-7) on the remodelling process known to be detrimental to the failing myocardium.

To follow on from Davie and McMurray [31], it would be worthwhile to recruit a much larger sample size to test the hypothesis that Ang-(1-7) has beneficial effects on haemodynamics in human heart failure.

Other cardioprotective effects that were demonstrated in animal studies might be translated into humans. We are planning to conduct a randomised controlled trial to test the hypotheses that Ang-(1-7) might improve cardiac hypertrophy and function.

Investigating the expression of Mas-gene mRNA or Mas-receptor distribution throughout the human failing heart will provide good insight into activity of Ang-(1-7). Furthermore, the study can also check if there is a correlation between expression and stage of HF and the remodelling process, ventricular size, and ejection fraction.

The nonpeptidic Ang (1-7) receptor agonist AVE0991 has already been shown to improve cardiac contractility in diabetic rats. Development of an oral nonpeptidic Ang (1-7) receptor agonist will enable us to perform a well-designed randomised controlled trial to study the cardioprotective effects of Ang-(1-7) in patients on ACE inhibitors or ARBs [81].

## 6. Conclusions

The main finding of this systematic review is that Ang-(1-7) plays an important cardioprotective role in heart failure in animals and in patients without heart failure. However, to date, the evidence for the role of Ang-(1-7) in human heart failure is limited. Nevertheless, accumulating evidence from a few studies demonstrated the importance of Ang-(1-7) as a potential therapeutic agent in heart failure.

## Figures and Tables

**Table 1:** Studies on human heart failure patients.

Study	Full publication or abstract	N	Endpoint	Key findings
Davie and McMurray [31]	Full	8	Blood pressure, forearm blood flow (FBF)	Ang-(1-7) has no significant effect on the endpoints.

Zisman et al. [12]	Full	22	Ang-(1-7) forming activity	Ang-(1-7) forming activity is increased in failing heart.

Campbell et al. [13]	Full	9	Ang-(1-7) levels in arterial and coronary sinus blood	39- and 22-fold increase of Ang-(1-7) levels in the coronary sinus and arterial blood, respectively (patients were receiving ACE inhibitor therapy). A parallel increase in Ang-(1-7) and Ang I levels was observed in HF patients on ACE inhibitors. A 7.5- and 2.25-fold increase in the Ang-(1-7)/Ang II ratio was associated with ACE inhibition in the coronary sinus and arterial blood, respectively.

Batlle Perales et al. [32]	Abstract	33 (end-stage HF); 11 (control)		Increased Mas receptor expression within myocardiums in the remodelling stage (as suggested by MMP3 and collagen I). Ang-(1-7) is therefore suggested to have a role in the remodelling process.

**Table 2:** Studies in non-heart failure patients.

Study	Full publication or abstract	Disease and control population (if applicable)	Study type, for example, cohort/biopsy from patients/human cells	N	Key endpoint(s)	Key finding(s)
Ueda et al. [18]	Full	Healthy; normotensive	Cohort	22	Forearm blood flow (FBF)	Ang-(1-7) attenuates Ang II-induced vasoconstriction.

Campbell et al. [13]	Full	Coronary artery disease (CAD)	Cohort	20	Ang-(1-7) levels in coronary sinus and arterial blood (key endpoint of relevance to Ang-(1-7) )	No significant change in the level of Ang-(1-7) after acute intravenous administration of an ACE inhibitor. On an assumed haematocrit of 45%, net Ang-(1-7) formation by the heart was unchanged after ACE inhibitor administration.

Ferrario et al. [33]	Full	Healthy volunteers and essential hypertension (HTN)	Cohort	Healthy volunteers (*n* = 31), essential HTN (*n* = 18)	Urinary concentration of Ang-(1-7)	Urinary Ang-(1-7) correlated inversely with arterial pressures reduced levels in untreated essential HTN patients.

Roks et al. [34]	Full	Patients undergoing coronary artery bypass grafting (CABG) surgery and healthy donors	Biopsies	CABG (*n* = 25), healthy donor (*n* = 1)	Plasma/tissue ACE activity and contractile function in human arteries	Ang-(1-7) blocks arterial vasoconstriction and inhibits ACE in plasma, atrial and arterial, tissues.

Wilsdorf et al. [19]	Full	Healthy subjects	Cohort	8	FBF; endothelial TPA release	Ang-(1-7) had no significant effect on the endpoints.

Pan et al. [35]	Full	NA	Primary cultures of human cardiac (HC) myocytes and HC fibroblasts	NA	Transcription of matrix metalloproteinase (MMP)−1, −2, and −9, and tissue inhibitors of matrix metalloproteinase (TIMP) −1, −2, and −3	Ang-(1-7) and Ang II have opposing and antagonistic effects. Ratios of MMPs to TIMPs were decreased (suggestive of less myocardial fibrosis) by Ang-(1-7).

Sampaio et al. [36]	Full	NA	Human aortic endothelial cells (HAECs)	NA	Activation of NAD(P)H oxidase; phosphorylation of extracellular signal regulated kinase (ERK)1/2; c-Src activation; SHP-2 phosphorylation; c-Src and SHP-2 interaction	Ang II signalling is counter-regulated by Ang-(1-7); effects are mediated probably via Mas.

Rajendran et al. [37]	Full	Normal subjects and patients with acute coronary syndrome (ACS)	Cohort study	Normal (*n* = 17) ACS (*n* = 17)	Platelet aggregation and responsiveness	Ang-(1-7) reduces platelet aggregation by potentiating sodium nitroprusside.

Peltonen et al. [38]	Full	Aortic regurgitation (AR); AR plus fibrosis (AR + F); aortic stenosis (AS); normal valve (control)	Cohort	Control (*n* = 11); AR (*n* = 14); AR + F (*n* = 20); AS (*n* = 61)	Expression of Mas receptor (key endpoint of relevance to Ang-(1-7))	Mas receptor mRNA levels in stenotic valves were lower than control, AR, and AR + F valves, further supporting the hypothesis that myocardial fibrosis is attenuated by Ang-(1-7), an endogenous Mas receptor agonist.

Christofi et al. [39]	Abstract	NA	Human saphenous vein cells (hSVSMC); human coronary artery cells (hCAC) and saphenous vein cells (hVTSM1)-derived immortalised human VSMC	NA	PCR on complimentary DNA expression of the Mas receptor (key endpoint of relevance to Ang-(1-7))	Mas gene expression was not detected in these cells.

Sampaio et al. [40]	Full	NA	Human aortic endothelial cells (HAECs)	NA	NO synthase (eNOS) expression/activity; role of Mas; regulation of ser1179 and thr495 phosphorylation sites of NO synthase; role of the phosphatidylinositol 3-kinase (PI3K)/Akt-pathway	HAECs express Mas and via this receptor mediate the activation of eNOS. eNOS activation and NO release by Ang-(1-7) involve the role of the PI3K/Akt pathway.

Lin et al. [41]	Full	NA	Human cardiac fibroblasts	NA	ACE2 mRNA and protein expression; phosphorylated ERK1/2 (p-ERK1/2) protein expression	Ang-(1-7) upregulates ACE2 expression, possibly independent of the Ang II-Angiotensin type 1 receptor signalling pathway. A positive feedback loop is observed (Ang-(1-7) increases ACE2 expression *in vitro*; increased ACE2 could promote more Ang-(1-7)).

Schindler et al. [42]	Full	Healthy young male nonsmokers	Cohort	8	Ang-(1-7) peptide level (determined relevant from the results section)	Irbesartan significantly increased Ang-(1-7) peptide levels. Atorvastatin had no effect on the Ang-(1-7) peptide level.

Rajendran et al. [43]	Full	NA	Whole blood	NA	Ang-(1-7) effect on NO responsiveness of platelets; is this associated with the modulation of O_2_^−^ release? Role of a specific Ang-(1-7) receptor	Effects of Ang-(1-7) occurred only in whole blood (another experiment was done on platelet-rich plasma). The antiaggregatory effects of the NO donor sodium nitroprusside (SNP) were potentiated by Ang-(1-7), probably by a specific Ang-(1-7) receptor. O_2_^−^ release suppression by SNP was potentiated by Ang-(1-7).

Ueda et al. [44]	Full	Healthy normotensive	Cohort	Procedure 1 (*n* = 8); procedure 2 (*n* = 8); procedure 3 (*n* = 6 out of 8 from procedure 2)	FBF	Ang-(1-7) in a dose-dependent manner potentiated the vasodilating effect of BK (doses of 1000 pmol/min and 100 pmol/min). Abolishing effects of an NO synthase inhibitor were not statistically significant. There was no effect of Ang-(1-7) on the vasodilating effects of either acetylcholine or SNP.

Gironacci et al. [45]	Full	NA	Mas receptor-yellow fluorescent protein (MasR-YFP) transfected human embryonic kidney 293T cells	Na	Relative cellular distribution of MasR-YFP	Ang-(1-7) causes the MasR to undergo endocytosis.

Luque et al. [46]	Full	Essential HTN	Cohort	24	Plasma concentration of Ang-(1-7) in the peripheral venous blood (key endpoint of relevance to Ang-(1-7))	The last dose of captopril (6 months after) produced significantly greater levels of Ang-(1-7). There was a negative correlation between plasma Ang-(1-7) and diastolic blood pressure in a subset of essential HTN subjects (in whom BP was controlled with captopril only).

Hayashi et al. [47]	Full	NA	Smooth muscle cells and endothelial cells	NA	ERK1/2 phosphorylation; smooth muscle cell proliferation; adhesion of monocytes to endothelial cells.	D-Ala (Mas antagonist) pretreatment decreased the inhibitory effect of olmesartan (in response to Ang II stimulation). Ang II increased the endpoints which olmesartan inhibited.

Zisman et al. [48]	Full	Orthotopic heart transplantation recipients (normal coronary anatomy and left ventricular function)	Cohort	4	^ 123^I-Ang-(1-7) was quantified in the myocardial circulation.	Ang-(1-7) is produced in the myocardial circulation. Reduced levels of Ang II (via enalaprilat) caused levels of Ang-(1-7) to decrease.

Gallagher and Tallant [49]	Full	NA	Lung adenocarcinoma cells; adenocarcinoma cells; squamous cell; carcinoma cells	NA	Cell number; DNA synthesis; inhibition time course of DNA synthesis by Ang-(1-7); Mas mRNA; DNa replication; ERK1/2 activities	Lung cancer cell growth is inhibited by Ang-(1-7), possibly via the activation of an Angiotensin peptide receptor, which may involve the ERK signal transduction pathway.

Pignone et al. [50]	Full	Systemic sclerosis (SSc); control subjects	Cohort	SSc (*n* = 32); control (*n* = 55)	Ang-(1-7), neutral endopeptidase (NEP), NO and prostaglandin I2 (PGI2) levels	Lower Ang-(1-7) levels were detected in patients with SSc. The Ang II/Ang-(1-7) ratio in SSc patients showed greater Ang II levels to Ang-(1-7). In the controls, Ang-(1-7) was prevalent. NEP, NO, and PGI2 levels are reduced in the SSc group.

Silva et al. [51]	Full	HTN (12 renovascular; 15 essential HTN); normotensive subjects; paediatric population	Cohort	Renovascular HTN (*n* = 12); essential HTN (*n* = 15); normotension (*n* = 32)	Ang-(1-7) levels in the blood (key endpoint of relevance to Ang-(1-7))	Ang-(1-7) levels are significantly higher in HTN (renovascular) patients compared to normal children. In the essential HTN subjects, Ang-(1-7) levels were significantly increased compared with normotensive subjects. Calcium channel blockers had no effect on the RAAS measurements.

Simões E Silva et al. [52]	Full	Normotensive healthy subjects; normotensive chronic renal failure (CRF); hypertensive CRF; end-stage renal disease (ESRD)	Cohort	Normotensive healthy subjects (*n* = 32); normotensive CRF (*n* = 23); hypertensive CRF (*n* = 34); ESRD (*n* = 21)	Radioimmunoassays for Ang-(1-7) levels (key endpoint of relevance to Ang-(1-7))	In the hypertensive CRF subjects, Ang-(1-7) levels were higher compared with normotensive CRF and healthy subjects. There was no difference between normotensive CRF and healthy subjects.

Nickenig et al. [53]	Full	NA	Human skin fibroblasts (from a skin biopsy; purchased)	NA		Ang-(1-7) competed for the Ang II binding, causing 80% loss of binding activity (approximately). Ang-(1-7) may be involved in DNA synthesis. Effects of Ang-(1-7) may be mediated via the Mas receptor.

Anton et al. [54]	Full	NA	Human umbilical vein endothelial cells (HUVECs)	NA	Tube formation of HUVECs	Ang-(1-7) exerted inhibitory effects on HUVEC tube formation. The effect of Ang-(1-7) was reversed by A779. Losartan also reversed the Ang-(1-7) mediated inhibition (similarly as to A779).

Villalobos et al. [55]	Abstract	NA	Cultured human vascular smooth muscle cells (HASMC)	NA	Levels of inducible NO synthase (iNOS) and NO release	Ang-(1-7) acts via Mas receptor and partially prevented vascular smooth muscle inflammation

Montezano et al. [56]	Abstract	NA	Cultured human microvascular endothelial cells (HMEC)	NA	Expression of a proinflammatory mediator, cell growth marker, and ET_*B*_receptor. ET_*A*_ receptor gene expression	Proinflammatory and mitogenic actions of ET − 1/ET_*A*_ receptor were negatively modulated by Ang-(1-7).

Peltonen et al. [57]	Abstract	Normal valves (control); AR; AR + F; AS	Cohort	Control (*n* = 11); AR (*n* = 11); AR + F (*n* = 17); AS (*n* = 57)	Expression of the Mas receptor in aortic valves (key endpoint of relevance to Ang-(1-7))	The Mas receptor is downregulated in stenotic aortic valves. Mas gene expression is not affected by statin treatment in the AS group.

Zhiming et al. [58]	Abstract	NA	THP-1 derived macrophages (human)	NA	mRNA and protein expression of ATP-binding cassette transporter A1 (ABCA1); macrophage cholesterol efflux	Decreased THP-1 induced macrophage cholesterol efflux, and ABCA1 expression by Ang II was reversed by Ang-(1-7) in a dose-response relationship. A-779 (Ang-(1-7) Mas receptor antagonist) had no effect on the endpoints.

Vzquez-Bella et al. [59]	Abstract	NA	Cultured human umbilical endothelial veins (HUVECs)	NA	Endothelial nitric oxide synthase (eNOS) and NO levels; L-arginine (eNOS substrate) asymmetric dimethylarginine (ADMA) (eNOS inhibitor); expression of ICAM-1 and VCAM-1 (cell adhesion molecules)	Ang-(1-7) pretreatment increased the NO release mediated by BK, an effect inhibited by A779 pre-treatment. Pretreatment with A779 increases levels of the inactive phosphorylated (Thr 495) form of eNOS and reduces the L-arginine/ADMA ratio. There was no effect of Ang-(1-7) on VCAM-1 and ICAM-1 expressions in nonstimulated (non-Ang II) HUVECs. There was reduced induction of ICAM-1 and VAM-1 in stimulated cells.

Santos et al. [60]	Abstract	NA	Confluent cultured human aortic and umbilical vein endothelial cells	NA	Formation of Ang-(1-7)	Generation of ^125^I-Angiotensin-(1-7) was time dependent when incubated with ^125^I-Angiotensin I.

Hermenegildo et al. [61]	Abstract	NA	Human umbilical vein endothelial cells (HUVECs)	NA	Expression of mRNA and protein of the enzymes associated with Ang-(1-7) production and NO synthesis	Oestradiol (E2) increased the expression of enzymes implicated in the production of NO and NO receptor expressions. A779 abolished E2's effect on NO synthase and NO receptor expression. A779 inhibited NO levels (NO levels increased by E2).

Kono et al. [62]	Full	Normal men and Bartter syndrome (BS)	Cohort	Normal (*n* = 5); BS (*n* = 3)	BP; aldosterone and plasma renin (key endpoint of relevance to Ang-(1-7))	A significant increase in BP was observed in normal men after Ang-(1-7) infusion. After the infusion of Ang-(1-7), it took 20 min (for systolic) and 30 min (for diastolic) for the pressor actions to cease. In patients with BS, Ang-(1-7) had no effect. Ang-(1-7) had no effect on aldosterone; however, it did lower plasma renin activity in both normal and BS groups. Ang-(1-7) has pressor actions, however, having effects <0.03% of Ang II.

Sasaki et al. [63]	Full	Essential HTN and normotensive controls	Cohort	Normotension (*n* = 8); essential HTN (*n* = 8)	FBF	Ang-(1-7) increases FBF through NO independent manner.

Calò et al. [64]	Full	Bartter syndrome/Gitelman syndrome* (BS/GS); normotensive healthy subjects; essential HTN	Cohort	BS/GS (*n* = 10); normotensive healthy subjects (*n* = 10); untreated essential HTN (*n* = 10)	Levels of ACE2 and Ang-(1-7)	Ang-(1-7) levels are elevated in BS/GS patients compared with the other two groups. In BS/GS patients, there was a direct correlation between ACE2 and Ang-(1-7). This study provides further support of the hypothesis that Ang-(1-7) regulates vascular tone.

*In Bartter syndrome/Gitelman syndrome, patients have gene defects in specific kidney transporters and ion channels, resulting in raised plasma Ang II and aldosterone, but intriguingly, they have normal or even low blood pressure. Their peripheral resistance is reduced, and they exhibit hyporesponsiveness to pressors.
